# The Art of Temperance in Eating

**Published:** 1858-06

**Authors:** 


### THE ART OF TEMPERANCE IN EATING.

Dear Doctor Hall:—Though not an "old patient" of yours, I have long been a reader of your Journal of Health, and have been so much interested in the first ten pages of your April number, that I feel an irresistible inclination to follow the example of your clerical correspondent, and send you a subscriber's answer to the question, "How are you?"

Leigh Hunt tells us, that Byron, in his latter days, "was in the habit of *taking too much* for his particular temperament—a fault, in one respect, the most pardonable in those who are the most aware of it; the *uneasiness of a sedentary stomach* tempting them to the very indulgence that is hurtful. Sometimes he would make an *outrageous dinner*, eating all sorts of things that were unfit for him, and suffering accordingly next day."

Jonathan Edwards, we see noting in his Diary: "I find that I cannot be convinced, in the time of eating, that to eat more would be to exceed the bounds of temperance, though I have had two years' experience of the like, and yet, three minutes after I have done, I am convinced of it. But yet again I overeat, thinking I shall be somewhat faint if I leave off then; but when I have finished, I am convinced again of excess, and so it is from time to time. *I have observed, that more really seems to be truth, when it is according to my inclination, than when otherwise.*"

Some years ago, the writer of this found his health failing, and all his efforts to recover it apparently rendered useless, from the fact that he was constantly "eating too much for his particular temperament," and that no resolutions made when suffering from the effect of one day's surfeit were sufficient to prevent his yielding to the tempations of the next day's table. "Vows made in pain, Ease would recant, as violent and void." Believing that this difficulty is a very common one, and that for this cause many noble minds among us are weak, and many sleep, he sends you a description of a method by which he was enabled at last, completely to break himself of this health-destroying habit. It is several years since he adopted it, and by the blessing of God, it has so effectually helped him, that he cannot remember that, during all that time, he has, in a single instance, passed the bounds of moderation in eating. While the complete power which it gives him over himself on all occasions, when for any reason special abstemiousness has been desired, is most delightful; and he hopes that other desponding dyspeptics may find in it a remedy for the worst half of their troubles.

Every Saturday, he prepares a slip of paper, of a size to be carried easily in the pocket, on which he marks the amount of food which he deems it desirable he should consume daily during the following week, in case he should perform no bodily labor.

In the winter, this amount is represented, as in a diagram, by twelve tally marks, each of which stands, in the writer's case, for an ordinary half slice of bread and butter, weighing about an ounce and a half, *or* an equivalent weight of any other food. With this check in his pocket, and a resolution to abide by it, he eats on Sunday morning as much as he desires for breakfast, usually about four half-slices of bread, or their equivalents; and as soon thereafter as he is alone, crosses off as many of the tally marks for Sunday as correspond with the amount he has eaten; never weighing his food, but measuring it with his eye, and being careful to cross off too many, rather than too few of the marks, where there is any doubt: at dinner, he does the same, taking care not to consume at that meal *all* of the balance of the twelve half-slices; and at supper, the uncrossed marks indicate the bounds beyond which he may not pass. And after the habit was once formed, he has never felt any disposition to pass it.

But sometimes he has to walk many miles during the day, and of course needs more food on that day than on the days in which he takes no exercise; and to provide for this necessity, he has left the two blank spaces at the foot of each day, in which he enters an extra mark for each half hour's walking, or other active bodily labor he performs on that day, to stand for the same amount of food as the other marks, and to be crossed out in the same way, when the food is eaten.

JEFFERSON declares that "no man ever repented eating too little;" and the writer can truly say, that he has known hundreds who were lamenting that their health, happiness, and usefulness were injured by the habit of eating too much; and he sincerely hopes, that any who have escaped from this melancholy thraldom, will not let any motives of false delicacy deter them from giving a history of their rescue. Our minds and our circumstances are so different, that what has proved such an easy remedy for the writer, may not avail for all.

He would add, that during several of the years preceding his escape from the habit of over-eating, he had taken hundreds of doses of medicine to remedy real and fancied ailments, but since he has, by means of this check, been able to say to the demands of appetite, "thus far and no further," he has generally had no occasion to take medicine at all, from the first day of January to the last day of December.

When a bad taste in the mouth in the morning, or any other indication, warns him, that all is not well within, he has but *to reduce materially* the number of his marks for the day or two to come, and the disagreeable symptoms generally vanish as if by magic. But *in no case* does he allow himself *to add* to the amount prescribed yesterday for to-day, except for exercise, as stated above. It is the quantity, rather than the kind of food, that kills so many of our students and professional men—though doubtless, the simpler the food the better.

ABERNETHY states, that he cured his indigestion and regained his flesh, by "going into the country, where he could get good milk and eggs, and living upon three ounces of baked custard, taken three times a day, with no drink but ginger-water. On this quantity of food he regained his flesh, and uniformly got better.

"An invalid may often, by judgment and self-control, enjoy a degree of 'high condition,' of which few men with unshattered constitutions know the luxury. But, for this, a most systematic and unvarying *attention to trifles* is required. The stomach is to be controlled, and watched like an experiment in chemistry." "If some demigod could regulate for mankind what they should eat and drink, he would put an end at one stroke to half the troubles which the world undergoes."

Yours, sincerely, M. J. N.

